# Integration of cytopathology with molecular tests to improve the lab diagnosis for TBLN suspected patients

**DOI:** 10.1371/journal.pone.0265499

**Published:** 2022-03-31

**Authors:** Abay Atnafu, Kassu Desta, Selfu Girma, Dawit Hailu, Gebeyehu Assefa, Shambel Araya, Dinksira Bekele, Liya Wassie, Kidist Bobosha

**Affiliations:** 1 Armauer Hansen Research Institute, Addis Ababa, Ethiopia; 2 Department of Medical Laboratory Science, Addis Ababa University, Addis Ababa, Ethiopia; Rutgers Biomedical and Health Sciences, UNITED STATES

## Abstract

**Background:**

Tuberculosis lymphadenitis (TBLN) diagnosis is often challenging in most resource poor settings. Often cytopathologic diagnosis of TBLN suspected patients is inconclusive impeding timely clinical management of TBLN suspected patients, further exposing suspected patients either for unnecessary use of antibiotics or empirical treatment. This may lead to inappropriate treatment outcome or more suffering of suspected patients from the disease. In this study, an integrated diagnostic approach has been evaluated to elucidate its utility in the identification of TBLN suspected patients.

**Methods:**

A cross-sectional study was conducted on 96 clinically diagnosed TBLN suspected patients, where fine needle aspirate (FNA) samples were collected at the time of diagnosis. FNA cytology, Ziehl-Neelsen (ZN), Auramine O (AO) staining, GeneXpert MTB/RIF and Real time PCR (RT-PCR) were performed on concentrated FNA samples. Considering culture as a gold standard, the sensitivity, specificity, positive and negative predictive values were calculated. Cohen’s Kappa value was used to measure interrater variability and level of agreement and a P-value of <0.05 was considered as statistically significant.

**Result:**

Out of the 96 FNA sample, 12 (12.5%) were identified to have *Mycobacterium tuberculosis* (*Mtb*) using ZN staining, 27 (28.1%) using AO staining, 51 (53.2%) using FNAC, 43 (44.7%) using GeneXpert MTB/RIF, 51 (53.1%) using Real time PCR (RT-PCR) and 36 (37.5%) using Lowenstein-Jensen (LJ) culture. Compared to LJ culture, the sensitivities of GeneXpert MTB/RIF, RT-PCR, and FNAC were 91.7%, 97.2%, and 97.2%, respectively and the specificities were 83.3%, 73.3%, and 68.3%, respectively. GeneXpert MTB/RIF and RT-PCR when combined with FNAC detected 61 (63.5%) cases as having *Mtb*, and the sensitivity and specificity was 100% and 58.3%, respectively.

**Conclusion:**

FNA cytology and RT-PCR detected more TBLN cases compared to other *Mtb* detection tools and the detection sensitivity even improved when FNA cytology was combined with GeneXpert MTB/RIF, performed on concentrated FNA sample, suggesting the combined tests as an alternative approach for improved diagnosis of TBLN.

## Introduction

Tuberculosis (TB) is generally caused by *Mycobacterium tuberculosis* (*Mtb*) complex and remains to be the major cause of death globally [1, 2]. The disease manifests either as pulmonary (PTB) or extrapulmonary (EPTB) forms, where TB lymphadenitis (TBLN) is the most frequent form of EPTB in Ethiopia [3, 4].

A number of laboratory diagnostic methods are available for the diagnosis of TBLN including direct smear microscopy using Ziehl-Neelsen (ZN) or Auramine O (AO) staining, culture, cytopathology examination of fine needle aspirates (FNA), molecular tests such as real-time polymerase chain reaction (RT-PCR) and GeneXpert MTB/RIF [5, 6].

However, clinical management is often challenged as a result of inconclusive cytopathology results, exposing the TBLN suspected patients for unnecessary use of antibiotics or empirical or delayed treatments. Unintended treatment outcomes also have a direct effect on the vulnerable patient, rendering them to experience severe clinical conditions including drug resistance and social and economic challenges. In this study, we evaluated the performance of integrating FNA cytology, ZN and AO staining, culture, GeneXpert MTB/RIF, and RT-PCR tests for diagnosis of TBLN using concentrated FNA samples. The finding from this study has strong implication on the utility of integrated use of laboratory tests on improved diagnosis of TBLN and to eventually increase the case detection rate of TBLN cases.

## Materials and methods

### Study setting

In this cross-sectional study, a total of 96 TBLN suspected patients were enrolled from the outpatient department (OPD) of the All-African Leprosy Research and Training (ALERT) Hospital, in Addis Ababa the between September 2020 and April 2021. Demographic and clinical data were collected using standardized questionnaire. FNA samples were collected from all TBLN suspected patients from sites with swollen lymph node: cervical, auxiliary, cervical and auxiliary or inguinal lymph node regions of the neck for cytologic examination at the Armauer Hansen Research Institute (AHRI) pathology laboratory, Addis Ababa. This study was approved by the ethics committee of the Addis Ababa University, Department of Medical Laboratory Sciences (DRERC) and the AHRI (Armauer Hansen Research Institute)/ALERT (All Africa Leprosy, Tuberculosis and Rehabilitation Training Center) Ethics Review Committee (AAERC). All participants gave written informed consent; for participants below 18 years, parental/guardian consent and child assent (for the age of 12–17) was obtained before enrolled into the study. All data were entered and analyzed using SPSS (Version 26, United states). The sensitivity, specificity, positive and negative predictive values were calculated using the culture results as the gold standard. Cohen’s Kappa value was used to measure inter-rater variability and level of agreement. A P-value < 0.05 was considered statistically significant.

### Laboratory testing procedure

FNA samples were collected and aliquoted for different laboratory testing as shown in S1 Fig. Briefly, a drop of the FNA aspirate was placed on a clean slide for direct smear cytomorphological analysis and left to air dry as previously described [7]. Another aliquot of the FNA sample was homogenized using sterile PBS, decontaminated using equal volumes of 3% sodium hydroxide, 2.9% Tri sodium citrate and 0.5% N-Acetyl-L-Cystein (NALC) and incubated for 15 minute at room temperature as previously described [8]. The incubation step was followed by centrifugation at 3000g for 15 minute, whereby the pellets were resuspended in phosphate buffered saline (PBS) solutions for use in Lowenstein-Jensen (LJ) culture and detection of acid fast bacilli (AFB) using standard Ziehl-Neelsen (ZN) or Auramine O (AO) staining techniques [9]. The growth on LJ culture media were checked regularly on weekly basis and those LJ tubes with no growth after eight weeks were reported as negative.

In parallel, GeneXpert MTB/RIF test was performed on concentrated and heat-inactivated samples following manufacturer’s instructions (Cepheid, United states of America). Briefly, 500μl of the FNA sample was mixed with 1.5 ml of the GeneXpert MTB/RIF diluents. Following 15minutes incubation, the whole mixture was transferred to the GeneXpert MTB/RIF cartridge and loaded onto GeneXpert machine (Cepheid DX system, version 4.8) for reading.

### DNA extraction

QIA amp mini kit (QIAGEN, Germany) was used for DNA extraction from heat inactivated FNA samples following the manufacturer’s protocol. In short, 20μl of QIAGEN proteinase K, 200μl of sample, and 200μl of buffer AL were added to a 2ml micro centrifuge tube and vortexed for 15 sec. The mixture was incubated for 10 minutes at 56°C. Ethanol (96%), 200μl, was added to the tube and mixed by pulse vortexing. The mixture was transferred to silica-based spin column without wetting the rim. Centrifugation proceeded at 6000xg for 1 minute. The elute was discarded and the spin column was washed by adding wash buffers AW1 and AW2 and centrifuging at 6000xg for 1min after adding each wash buffer. Finally, the spin column was dried by Centrifuging at 20000 xg (14000 rpm) for 3 minutes. After placing spin column into a clean 1.5ml micro centrifuge tube, 50μl Buffer AE was added and incubated at room temperature (25°C) for 1 minute and then centrifugation at 6000xg (8000 rpm) for 1 to elute the DNA.

### IS1081 typing by real-time PCR

The number of IS1081 copies is stable–Six copies–between isolates and species within M. Tuberculosis complex. With six stable copies of IS1081 in these species, the IS1081 becomes a good tool for detection of M. Tuberculosis complex genome in clinical samples. The following IS1081 primers were used:

10uM IS1081_Fw 5’-GATCCTTCGAAACGACCA-3’
10uM IS1081_Rev 5’- CGGTGTCGATAAGATGAGA-3’
10uM IS1081_Probe [6FAM]-CGAAGGAAATGACGCAATGACCTC-[BHQ1]

Real time to detect MTB complex in DNA extracted from FNA sample.

### Master mix preparation

Master Mix for 1X sample was prepared by mixing 2.5 μl of IS1081f and IS1081r, 0.5 μl Probe FAM labeled, 12.5 μl hot start master mix, and 2 μl molecular grade water in a biosafety cabinet, an aliquot of 20ul of the master mix was added into each Real time-PCR reaction tube. DNA templates i.e. (controls and unknown samples) were added to each tube containing the master mix.

The tubes were placed in a Rotor Gene 3000 Real Time PCR machine (Corbett research, Australia) and run with thermal conditions of initial denaturation at 95°C for 15 mins, and 40 cycles of 95°C for 15 sec, 58°C for 1 min. Samples were considered positive if Ct value was less than 36.

## Results

### Distribution of lymph nodes and cytopathological findings

Of all FNA samples processed, 51 (53.1%) were aspirates mixed with blood, 36 (37.5%) purulent and the remaining 9 (9.4%) were aspirates with cheesy-like material. Depending on the site of swollen lymph node, the majority of the lymph nodes were from cervical region (83.3%), followed by auxiliary (9.4%) and inguinal (6.3%). A small proportion of the lymph nodes (1.04%) were also from cervical and auxiliary regions. Based on cytological examinations, 53.1% (51) of the FNA samples suggested *Mtb* infection. Of these 51 positive samples, 44.8% of the FNA samples were collected from cervical site and the 1% was that collected from cervical and auxiliary areas Table 1.

**Table 1:** FNA sample collection sites and corresponding cytopathological findings.

		Cytopathology finding			Total
		Positive	Negative	Inconclusive	
Site of FNA sample collection	Cervical	43	34	3	80
	Axillary	5	4	0	9
	Cervical & axillary	1	0	0	1
	Inguinal	2	4	0	6
Total		51	42	3	96

### Distribution of FNAC positive smear to various group of cytomorphological pattern

A Higher number of FNAC positive smear was observed in group 1, whereas the least number was observed in last group Table 2.

**Table 2:** FNAC positive smear distribution against cytomorphological pattern.

Cytomorphological pattern	FNAC Smear positive	P value
Epithelioid granuloma with necrosis (Group 1)	23	<0.001
Epithelioid granuloma without necrosis (Group 2)	10	
Necrosis with polymorphonuclear leukocyte (Group 3)	12	
Necrosis only (group 4)	6	

### Comparative analyses of the different diagnostic methods

Taking culture as a gold standard, the sensitivities and specificities of the assays were compared (Table 3). Overall, 36 (37.5%) samples yield *Mtb* growth on LJ culture media out of the 96 FNA samples collected, where the colony characteristics ranged from single colony to multiple colonies. Four samples got contaminated and were excluded from the analysis. The average growth period for LJ culture ranged between 3 and 6 weeks. Fine needle aspirate cytology, RT-PCR and GeneXpert MTB/RIF detected more *Mtb* bacilli (51, 51, and 43 respectively) compared to the microscopic staining techniques (Zn (12) and AO (27)) (Table 3); however, their overall agreement with LJ culture ranged from poor with ZN staining (κ-value = 0.363) to moderate with AO staining (κ-value = 0.767).

**Table 3:** Comparative detection of *Mtb* using different TB laboratory diagnostic methods against LJ culture.

		LJ Culture		Kappa Value	P value
		Positive	Negative		
ZN	Positive	12	0	0.385	<0.001
	Negative	24	59		
AO	Positive	27	0	0.789	<0.001
	Negative	9	59		
Xpert	Positive	33	10	0.722	<0.001
	Negative	3	50		
RT PCR	Positive	35	16	0.651	<0.001
	Negative	1	44		
FNAC	Positive	35	16	0.605	<0.001
	Negative	1	41		
	Inconclusive	0	3		

**Abbreviations**: LJ = Lowenstein-Jensen; ZN = Ziehl-Neelsen staining; AO = Auramine O staining; RT-PCR = Real time-polymerase chain reaction; FNAC = Fine needle aspirate cytology

According to an earlier report [10], a positive smear grading for FNAC has been categorized as epithelioid granuloma with (Group 1) or without (Group 2) necrosis, necrosis with polymorphonuclear leukocyte (Group 3) or caseous necrosis (Group 4). The sub grouping help to increase the sensitivity of FNAC as a given smear usually doesn’t exhibit all four patterns at the same time. Accordingly, a smear exhibiting any one of these cytopathology diagnostic features were reported as positive, tubercular lymphadenitis, or cold abscess and of the 96 FNA samples analyzed, 51 (53.1%) were detected as positive by FNAC. The FNAC positivity rate in each sub group was also compared with LJ culture, GeneXpert MTB/RIF and RT-PCR, where their agreement ranged from 0.6 to 0.711 **(**Table 4**)**.

**Table 4:** Comparative analyses of *Mtb* detection between cytomorphological patterns and LJ culture, RT-PCR, and GeneXpert.

Methods		Cytomorphological pattern				Kappa Value	P Value
		Group 1	Group 2	Group 3	Group 4		
LJ Culture	Positive	23	2	8	2	0.605	<0.001
	Negative	0	8	4	4		
RT PCR	Positive	23	5	11	5	0.711	<0.001
	Negative	0	5	1	1		
GeneXpert	Positive	23	4	9	2	0.6	<0.001
	Negative	0	6	3	4		

Previous history of anti-TB treatment didn’t show significant association with GeneXpert MTB/RIF resistance. None of the participants showed resistance to rifampicin as detected by GeneXpert.

The detection rate of all parameters included in this study as compared with FNAC was summarized as shown in Table 5.

**Table 5:** Summary of detection rate of ZN, AO, GeneXpert, and RT PCR against FNAC.

		Cytopathology			Total	P Value	Kappa Value
		Negative	Positive	Inconclusive			
ZN	**Negative**	41	40	3	84	0.004	0.168
	**Positive**	1	11	0	12		
AO	**Negative**	41	25	3	69	<0.0001	0.437
	**Positive**	1	26	0	27		
GeneXpert	**Negative**	38	13	2	53	<0.0001	0.6
	**Positive**	4	38	1	43		
RT PCR	**Negative**	36	7	2	45	<0.0001	0.675
	**Positive**	6	44	1	51		

ZN and AO staining have detected 1 additional positive case which was missed by FNAC. From all FNAC positives cases, 11 of them were also positive with ZN. While AO detected 26 positive cases from all FNAC positive results.

Of the 42 cytology negative results, 4 were positive with GeneXpert MTB/RIF assay and 13 cases were negative by GeneXpert MTB/RIF from all FNAC positive cases. RT PCR detected additional 6 positive cases which were FNAC negative. Among the 3 inconclusive results, 1/3 was positive with both RT PCR and GeneXpert MTB/RIF assay while 2/3 were negative.

Positivity rate of molecular methods were compared with that of FNAC. RT PCR detected 6 positive cases which were FNAC negative and 9 GeneXpert negative cases as positive. GeneXpert also detected 4 FNAC negative cases as positive and 1 RT PCR negative case as positive. FNAC in turn detected 7 positive cases missed by RT PCR and 13 negative cases by GeneXpert were positive by FNAC.

### Diagnostic performances of ZN, AO, GeneXpert, RT PCR, and FNAC

The Sensitivity, specificity, positive predictive value, negative predictive value, and positive and negative likelihood ratio were calculated with 95% confidence interval with culture being gold standard. S1 Table.

The sensitivity of GeneXpert MTB/RIF assay, 91.7% [95%CI (91.8%– 100%)], was slightly lower than RT PCR, the specificity, 83.3% [95%CI (73.9%– 92.8%)], was higher when compared with that of RT PCR. The sensitivity, specificity, positive predictive value, and negative predictive value of cytology were 97.2% [95%CI (91.8%– 100%)], 68.3% [95%CI (60%– 82.5%)], 68.6% [95%CI (55.8%– 81.3%)], and 97.6% [95%CI (93%– 100%)] respectively. S1 Table.

### Diagnostic performance of combined methods

The molecular methods (GeneXpert and RT PCR) were combined together with FNAC to see if the laboratory diagnosis of TBLN can be improved. As shown in the table, the three methods in combination detected 61 positive cases. The level of agreement with FNAC (Kappa value) was 0.772. S2 Table.

To see the combination effect of the molecular methods separately, GeneXpert MTB/RIF assay with FNAC, and RT PCR with FNAC were compared with the result from FNAC. Kappa value GeneXpert assay with FNAC was 0.856 with P value of <0.001. Similarly, the kappa value was 0.815 with p value of <0.001 for combined effect of RT PCR with FNAC. S2 Table.

The detection rate of combined methods (Molecular methods + FNAC), GeneXpert + FNAC, and RT PCR + FNAC were calculated in comparison with culture. The detection rate when GeneXpert+RT PCR+FNAC combined together was higher prevailing 61 positive cases. A substantial agreement was shown when GeneXpert was combined with FNAC. The sensitivity, specificity, PPV, NPV, level of agreement, and P value of combined method (FNAC+RT PCR+GeneXpert), GeneXpert + FNAC, and RT PCR + FNAC were calculated in comparison with culture (gold standard). The sensitivity of GeneXpert+RT PCR+FNAC, GeneXpert+FNAC, and RT PCR+ FNAC was 100% while the specificity of Xpert+FNAC, 66.7%, was relatively higher. S3 Table.

## Discussion

Diagnosis of TBLN in Ethiopia mainly relies on FNAC. The method was proven to be simple, sensitive, and inexpensive but often times, it is based mainly on suggestive features of tuberculosis such as epithelioid granuloma and caseous necrosis rather than depending on the direct detection of bacteria which might occur due to factors other than Tuberculosis and may produces inconclusive results in some instances [11]. Some reports have shown that combining FNAC with other diagnostic methods such as bacteriological method and molecular methods such as PCR and GeneXpert may alleviate the problem associated with the non-specific nature of FNAC and improve the diagnostic index [11–13].

The time-consuming nature of culture was also noted well with the current study. The earliest growth recorded in this study was three weeks and the longest period being six weeks. In this study, Culture has shown a positive detection rate of 37.5% of the total participants. Among FNAC positive cases 70.5% of them were also positive with culture. The remaining positive cases missed by culture from all FNAC positive cases might reflect the non-specific nature of FNAC. Same finding has been shown by Kant *et al* [14]. Among the GeneXpert positive cases, 76.6% were also positive by culture. In contrast, Tamana *et al* has shown the detection rate of culture from all GeneXpert positive cases was 44.3% [15]. Based on kappa statistics analyzed in this study, GeneXpert MTB/RIF assay relatively has better agreement with culture having a kappa value of 0.72 when compared with level of agreement of culture with RT PCR and FNAC. The kappa value reported in this study was much higher than that of the report made by Tamana *et al* [15]: a kappa value of 0.39 showing a relatively poor agreement between GeneXpert and culture when compared with this study. This might be due to the fact that in this study Culture, GeneXpert MTB/RIF assay and other methods were performed on the same concentrated sample after decontamination process has been carried out.

Culture method also detected positive cases of 28.6% from all ZN negative cases. Zewdie et al observed an inconsistent finding with 8.7% positivity of culture method from all ZN negative cases [16]. In both studies, concentration method has been performed, but the sensitivity of ZN is much lower in this study when compared with the finding from Zewdie et al. The lower bacterial load might have contributed for the lower sensitivity of ZN method in this study. ZN and AO staining methods provide results in a simple, quick and inexpensive way as compared to culture. The problem especially when it comes to diagnosing FNA samples is the low sensitivity of the two staining methods although the sensitivity of AO is higher than ZN. AO is better not only in sensitivity but also in the level of agreement with culture method as it is higher than ZN.

This study aimed at integrating the molecular methods: GeneXpert MTB/RIF assay and RT PCR with FNAC to improve the laboratory diagnosis of TBLN patients. GeneXpert MTB/RIF assay is a simple, sensitive, and automated method endorsed by the WHO. The method relied on the identification of rpoB gene associated with RIF drug resistance. RT PCR assay in this study targets IS1081 gene present in all MTBC and in a stable copy number of 5–7 repeats per genome [17, 18]. The finding from this study showed the prevalence of positivity of 44.5% and 53.1% for GeneXpert and RT PCR respectively. Report from Tadesse *et al* showed increased positivity of GeneXpert, that is 60.1%, in comparison with the present study [19]. In this study, there were FNA samples mixed with blood which might have contributed for the sample to be of lower quality and for the GeneXpert positivity to be lower.

When compared with culture, GeneXpert has detected an additional 10 (16.67%) positive cases which were culture negative. This finding showed a slight similarity with Samreen *et al* who reported 38 (19.6%) additional positive cases by GeneXpert from culture negative cases [20]. The kappa value of the two molecular methods has been analyzed against culture, and GeneXpert assay has a better agreement with culture having a kappa value of 0.72 and RT PCR with a kappa value of 0.65, with a little lower agreement when compared with GeneXpert.

GeneXpert and RT PCR have detected 4 (7.8%) and 6 (11.7%) additional positive cases respectively which were negative with FNAC. Tadesse *et al* reported 15.6% additional positive cases by GeneXpert which did not have suggestive feature of TBLN by FNAC (19). Among all FNAC positive cases, 74.5% were also positive with GeneXpert MTB/RIF assay and 86.2% were also positive with RT PCR assay. Regarding level of agreement with FNAC, both molecular methods showed an agreement with a kappa value of 0.6 and 0.675 for GeneXpert and RT PCR respectively.

In contrast to the finding from Mengistu et al, which reported a sensitivity and specificity of 78% and 74% for GeneXpert MTB/RIF assay [21], the present study showed a higher sensitivity and specificity of 91.7% and 83.3% respectively. The increased sensitivity and specificity observed in this study may have resulted from the application of concentration method. The sensitivity in the present study was also higher than what has been reported by Mulualem et al where GeneXpert MTB/RIF assay was performed from a concentrated FNA sample but having a sensitivity of 87.8% which was closer but still lower to what has been shown in this study [22].

The sensitivity and specificity of RT PCR in this study was found to be 97.2% and 73.3%. This finding is not consistent with Babafemi *et al*, which showed a sensitivity of 70% and higher specificity of 99% [23]. A very low sensitivity of 17.1% and a specificity of 100% was also reported by Linasmita *et al* [24] which happens to be higher than that of the specificity observed in the present study. The reason for the big difference seen in the two studies might be due to the difference of primer set used in both studies.

FNAC has a sensitivity of 97.2% and a specificity of 68.3%. Tamana E-Nur also reported the same pattern of FNAC having a higher sensitivity and lower specificity. The finding from Tamana E-Nur showed the sensitivity of 79.7% and specificity of 48.1% [25]. This seems to be lower than the finding from this study. Upadhyay, and Thakker also reported a sensitivity of 90.9% and specificity of 67.2% [26] showing a similar decrease in specificity with the present study.

A study by Manitchotpisist *et al* has shown that the combined use of PCR with FNAC was helpful in improving the diagnosis of TBLN. They have shown how the sensitivity and specificity of FNAC can be improved from 48% and 87.5% to 84% and 100% respectively [27]. The molecular tools have been combined with FNAC in this study to see how the combination may improve the diagnostic limitation of FNAC observed so far. Initially, each molecular tool was combined with FNAC and the level of agreement was assessed with FNAC. Sensitivity, Specificity, PPV, and NPV were calculated using culture as gold standard. When FNAC was combined with GeneXpert MTB/RIF assay (FNAC+GeneXpert MTB/RIF assay), it has a substantial agreement with a kappa value of 0.856. This finding was very close with the kappa value calculated for the combination of FNAC with RT PCR (FNAC+RT PCR) which showed a kappa value of 0.815. GeneXpert+FNAC showed a slightly better capacity in terms of accuracy and precision. The sensitivity, specificity, PPV, and NPV showed a slight increment when compared with what was observed in FNAC+RT PCR.

## Conclusion

We concluded that the combination of molecular methods, performed on concentrated FNA sample, with FNAC observed in this study clearly showed how the diagnostic value could be enhanced to an improved state than that observed in FNAC alone. Especially combining FNAC with GeneXpert MTB/RIF assay generated a very useful result in delivering affirmative result hence initiating early and appropriate treatment, and in providing better health care service.

## Recommendation

We recommend that easily accessible, simple and rapid diagnostic methods such as GeneXpert MTB/RIF assay and RT PCR, performed on concentrated FNA sample, can be used to improve the diagnostic challenge of tubercular lymphadenitis, and we also recommend country wide studies to be performed which would give a better picture regarding the application of molecular tests in combination with FNAC, and the challenges seen in the diagnosis of Tubercular lymphadenitis.

## Supporting information

S1 Fig (TIF)

S1 Table Diagnostic performance of ZN, AO, Xpert, RT PCR, and FNAC. (DOCX)

S2 Table Detection rate and level of agreement of combination of molecular methods against FNAC. (DOCX)

S3 Table Diagnostic performance of combined methods. (DOCX)

S1 Dataset (XLSX)
