# Supplementary figures and images for: Integration of cytopathology with molecular tests to improve the lab diagnosis for TBLN suspected patients
Source: PLoS One. 2022 Mar 31;17(3):e0265499. doi: 10.1371/journal.pone.0265499 (PMC8970391; doi:10.1371/journal.pone.0265499)

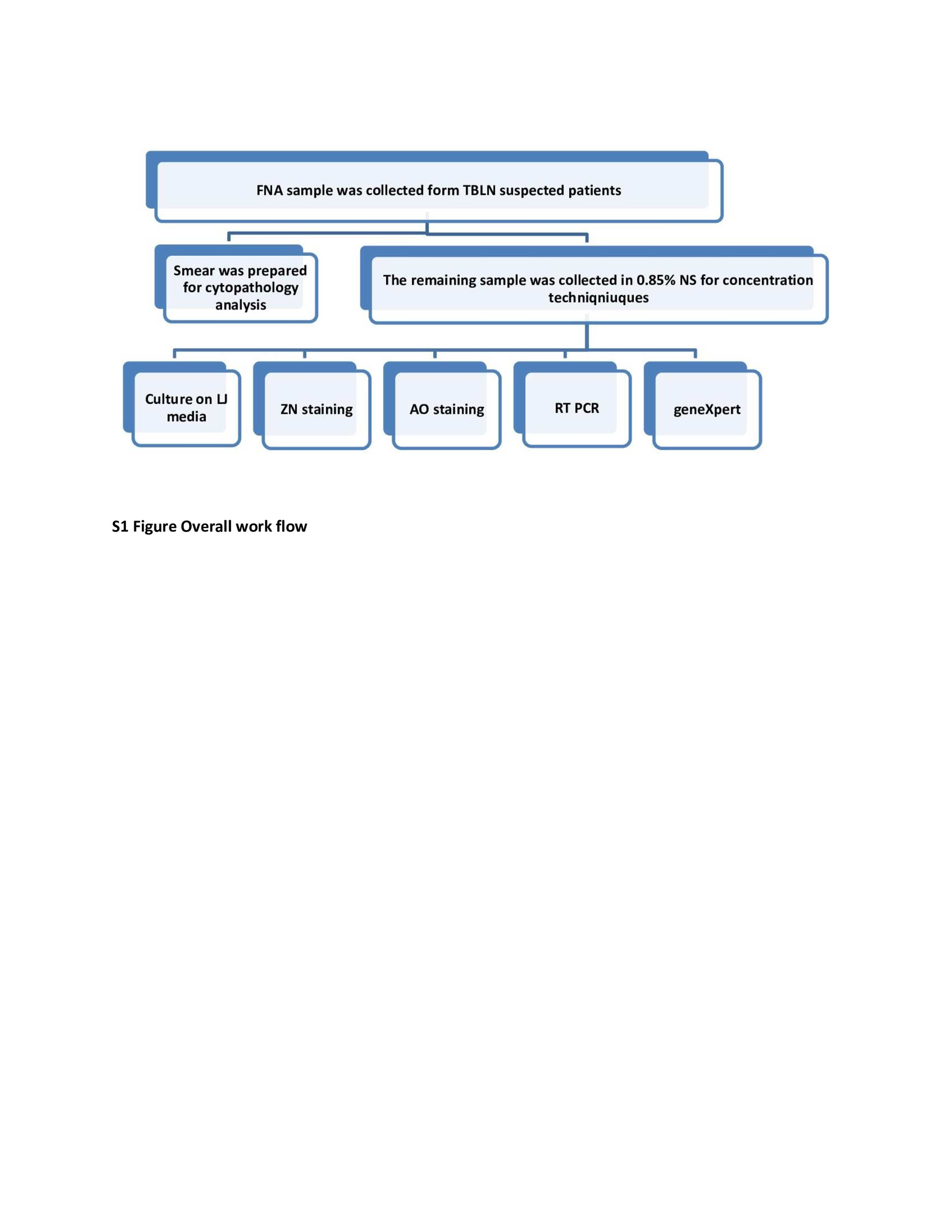

Supplement: S1 Fig — (TIF) [file pone.0265499.s001.tif]
