# Supplementary material for: Integration of cytopathology with molecular tests to improve the lab diagnosis for TBLN suspected patients
Source: PLoS One. 2022 Mar 31;17(3):e0265499. doi: 10.1371/journal.pone.0265499 (PMC8970391; doi:10.1371/journal.pone.0265499)
Supplement: S1 Table — (DOCX) [file pone.0265499.s002.docx]

S1 Table Diagnostic performance of ZN, AO, Xpert, RT PCR, and FNAC

|  | **ZN** | **AO** | **GeneXpert** | **RT PCR** | **FNAC** |
| --- | --- | --- | --- | --- | --- |
| **Sensitivity** | 33% | 75% | 91.70% | 97.20% | 97.20% |
| **95% CI** | 17.9% - 48.7% | 60.8% - 89.2% | 91.8% - 100% | 91.8% - 100% | 91.8% - 100% |
| **Specificity** | 98.3% | 98.3%% | 83.30% | 73.30% | 68.3% |
| **95% CI** | 95.1% - 100% | 95.1% - 100% | 73.9% - 92.8% | 62.1% - 84.5% | 60% - 82.5% |
| **PPV** | 92.3% | 96.4% | 76.70% | 68.60% | 68.6% |
| **95% CI** | 77.8% - 100% | 89.5% - 100% | 64.1% - 89.3% | 55.9% - 81.3% | 55.8% - 81.3% |
| **NPV** | 71.10% | 86.8% | 94.30% | 97.80% | 97.60% |
| **95% CI** | 61% - 81.8% | 78% - 95% | 88% - 100% | 93.5% - 100% | 93.4% - 100% |
| **P Value** | <0.001 | <0.001 | <0.001 | <0.001 | <0.001 |
| **PLR** | 20 | 45 | 5.5 | 3.6 | 3.1 |
| **95% CI** | 2.71-147 | 6.4 - 317 | 3.1 – 9.8 | 2.4 – 5.6 | 2.1 – 4.5 |
| **NLR** | 0.68 | 0.25 | 0.1 | 0.04 | 0.041 |
| **95% CI** | 0.54 – 0.86 | 0.14 – 0.44 | 0.03 – 0.3 | 0.005 – 0.26 | 0.006 – 0.3 |
