# Supplementary material for: Integration of cytopathology with molecular tests to improve the lab diagnosis for TBLN suspected patients
Source: PLoS One. 2022 Mar 31;17(3):e0265499. doi: 10.1371/journal.pone.0265499 (PMC8970391; doi:10.1371/journal.pone.0265499)
Supplement: S2 Table — (DOCX) [file pone.0265499.s003.docx]

S1 Table Detection rate and level of agreement of combination of molecular methods against FNAC

|  | | **Cytopathology** | | | **Total** | **Kappa Value** | **P value** |
| --- | --- | --- | --- | --- | --- | --- | --- |
|  |  | Negative | Positive | Inconclusive |  |  |  |
| **Xpert+FNAC+RT PCR** | Negative | 34 | 0 | 1 | 35 | O.772 | <0.001 |
|  | Positive | 8 | 51 | 2 | 61 |  |  |
| **XPERT+FNAC** | Negative | 38 | 0 | 2 | 40 | 0.856 | <0.001 |
|  | Positive | 4 | 51 | 1 | 56 |  |  |
| **RT PCR+FNAC** | Negative | 36 | 0 | 2 | 38 | 0.815 | <0.001 |
|  | Positive | 6 | 51 | 1 | 58 |  |  |
