# Supplementary material for: Integration of cytopathology with molecular tests to improve the lab diagnosis for TBLN suspected patients
Source: PLoS One. 2022 Mar 31;17(3):e0265499. doi: 10.1371/journal.pone.0265499 (PMC8970391; doi:10.1371/journal.pone.0265499)
Supplement: S3 Table — (DOCX) [file pone.0265499.s004.docx]

S1 Table Diagnostic performance of combination of molecular methods with FNAC against gold standard

|  | | Culture | | | **Total** | **Kappa** | **P Value** |
| --- | --- | --- | --- | --- | --- | --- | --- |
|  |  | Negative | | Positive |  |  |  |
| RT PCR+XPERT+FNAC | Negative | 35 | | 0 | 35 | 0.512 | <0.001 |
|  | Positive | 25 | | 36 | 61 |  |  |
| **Sensitivity** | 100% [95% CI(100%)] | | | | |  |  |
| **Specificity** | 58.3 % [95% CI(45.8% - 70.8%)] | | | | |  |  |
| **PPV** | 59% [95% CI(46.7% - 71.3%)] | | | | |  |  |
| **NPV** | 100% [95%CI (100%)] | | | | |  |  |
| XPERT+FNAC | Culture | | | | **Total** | **Kappa** | **P Value** |
|  |  | Negative | | Positive |  |  |  |
|  | Negative | 40 | | 0 | 40 | 0.60 | <0.001 |
|  | Positive | 20 | | 36 | 56 |  |  |
| **Sensitivity** | 100 % [95% CI (100%)] | | | | |  |  |
| **Specificity** | 66.7% [95% CI (54.7% - 78.6%)] | | | | |  |  |
| **PPV** | 64.3% [95% CI(51.7% - 76.8%)] | | | | |  |  |
| **NPV** | 100% [95% CI (100%)] | | | | |  |  |
| RT PCR+XPERT | Culture | | | | Total | Kappa | P value |
|  |  | Negative | Positive | |  |  |  |
|  | Negative | 38 | | 0 | 38 | 0.564 | <0.001 |
|  | Positive | 22 | | 36 | 58 |  |  |
| **Sensitivity** | 100% [95% CI (100%)] | | | | |  |  |
| **Specificity** | 63.3% [95% CI (51.1% - 75.5%)] | | | | |  |  |
| **PPV** | 62.1% [95% CI (49.5% - 74.5%)] | | | | |  |  |
| **NPV** | 100% [95% CI (100%)] | | | | |  |  |
